# Electroconvulsive therapy modulates the interplay between depressive symptoms in difficult-to-treat depression: A longitudinal network analysis

**DOI:** 10.1192/j.eurpsy.2025.10052

**Published:** 2025-06-16

**Authors:** Marialaura Lussignoli, Marco Bortolomasi, Giulia Perusi, Giorgio Pigato, Alessandra Minelli, Fabio Sambataro

**Affiliations:** 1 Department of Neuroscience (DNS), University of Padova, Padua, Italy; 2 Padova Neuroscience Center, University of Padova, Padua, Italy; 3 Psychiatric Hospital “Villa Santa Chiara”, Verona, Italy; 4 Department of Molecular and Translational Medicine, University of Brescia, Brescia, Italy; 5 Psychiatry Clinics, Padua University Hospital, Padua, Italy; 6 Genetics Unit, IRCCS Istituto Centro San Giovanni di Dio Fatebenefratelli, Brescia, Italy

**Keywords:** Bipolar disorders, Difficult-to-treat depression, Electroconvulsive therapy, Major Depressive Disorder, network analysis

## Abstract

**Background:**

Difficult-to-treat depression (DTD) is a common clinical challenge for major depressive disorder and bipolar disorders. Electro convulsive therapy (ECT) has proven to be one of the most effective treatments for this condition. Although several studies have investigated individually the clinical factors associated with the DTD response, the role of their interplay in the clinical response to ECT remains unknown. In the present study, we aimed to characterize the network of symptoms in DTD, evaluate the effects of ECT on the interrelationship of depressive symptoms, and identify the network characteristics that could predict the clinical response.

**Methods:**

A network analysis of clinical and demographic data from 154 patients with DTD was performed to compare longitudinally the patterns of relationships among depressive symptoms after ECT treatment. Furthermore, we estimated the network structure at baseline associated with a greater clinical improvement (≥80% reduction at Montgomery–Åsberg Depression Rating Scale total score).

**Results:**

ECT modulated the network of depressive symptoms, with increased strength of the global network (*p* = 0.03, Cohen’s *d* = −0.98, 95% confidence interval = [−1.07, −0.88]). The strength of the edges between somatic symptoms (appetite and sleep) and cognitive-emotional symptoms (tension, lassitude, and pessimistic thoughts) was also increased. A stronger negative relationship between insomnia and pessimistic thoughts was associated with a greater improvement after ECT. Concentration difficulties and apparent sadness showed the greatest centrality.

**Conclusions:**

In conclusion, ECT treatment may affect not only the severity of the symptoms but also their relationship; this may contribute to the response in DTD.

## Introduction

Depression is one of the most important causes of disability and a major contributor to the global burden of disease in the world [1], with a lifetime prevalence that could reach up to 30% in the general population [2]. Its clinical presentation is highly heterogeneous, encompassing several symptoms that extend beyond affect and mood disturbance. These include neurocognitive impairment, altered vegetative functions, and psychomotor abnormalities, all of which can significantly contribute to disability. Although antidepressant treatments are available, difficult-to-treat depression (DTD) is a common challenge, which considerably complicates its clinical management [3]. DTD reaches a prevalence of 19% [4] and it can also occur in major depressive disorder (MDD) and in bipolar disorder (BD).

Several pharmacological, somatic, and psychotherapeutic approaches have been proposed for the treatment of DTD [5]. Electroconvulsive therapy (ECT) remains one of the most effective treatments, with an estimated efficacy of 48% in nonpsychotic depression and higher in patients with psychotic symptoms [6, 7]. ECT has differential effects on depressive symptoms. Among remitters, core mood/anhedonia symptoms showed greater improvement than somatic and insomnia dimensions [8]. Although differential trajectories of symptom clusters after ECT have been studied, their interrelations are still understudied.

To study the relationship between symptoms in psychiatric disorders, network analysis, a relatively novel analytical approach, has been proposed [9]. According to network theory, mental illnesses may be described by the interaction of symptoms within a network, whereby the occurrence of one symptom increases the probability that an interrelated set of symptoms will also manifest [10]. A general property of a network is density, which describes the number of connections between nodes: the higher it is, the greater the interconnection between the symptoms and their co-occurrence. This relationship can determine clusters of psychopathology that reinforce each other and ultimately become self-sustaining. In highly interconnected networks, the presence of a central symptom can lead to a cascade activation of a series of symptoms that persist even after the disappearance of the triggering factor [11]. This mechanism is thought to support chronicity, whereby self-sustaining feedback loops triggered could lead to a worsening of the psychopathological condition and resistance to the treatment of severe mental illness [9, 12–15]. The network analysis approach has been used in several psychiatric disorders, including MDD. In these studies, the authors demonstrated that the psychopathological network changes with depression treatment [16]. They also identified central symptoms (primarily fatigue and depressed mood) that could play a key role in determining the chronicity of the pathology [16, 17] and, more specifically, a depressed mood emerged as a plausible driving symptom for activating other symptoms [18]. For these reasons, the network analysis in DTD could allow us to understand which symptoms may be associated with a poor response to treatment, chronicity, and a negative prognosis. Understanding the symptoms related to chronicity and hypothesizing how to treat them could lead to improved care.

The present study aimed to characterize the network of symptoms in patients with DTD and evaluate the effects of ECT on the interrelationship of depressive symptoms. Furthermore, we wanted to understand whether the baseline network of symptoms could predict the effectiveness of ECT. We expected that the depressive symptom network may be modified after ECT, and that central symptoms would differ between patients with better responses and those with poorer outcomes. Based on previous literature, we expected fatigue and depressed mood to play a key role in the development of chronicity in a depressive episode.

## Materials and methods

Adult inpatients of the Psychiatric Ward of Villa Santa Chiara (Verona, Italy) admitted from 2003 to 2022, with a diagnosis of DTD in MDD or BD, according to the Diagnostic and Statistical Manual of Mental Disorder IV-TR [19], were defined as “depression that continues to cause significant burden despite usual treatment efforts” [20]. Patients were excluded if they had an organic brain disorder or a diagnosis of intellectual disability. All participants signed a written informed consent form for the general use of their data for research purposes, anonymously and in an aggregate form. In accordance with the local Internal Review Board, the passive review of medical records for this retrospective and naturalistic research study did not require patients to provide further informed consent. This study was carried out by following the guidelines of the Declaration of Helsinki of 1964 [21].

Patients were maintained on stable pharmacological treatment for at least 3 weeks before ECT, and any medications that reduced ECT efficacy (e.g., benzodiazepines) were reduced or stopped based on clinical judgment. Pharmacological treatment was recorded in terms of defined daily dose equivalents [22] and the number of psychotropic drugs prescribed for each class. Depression severity was assessed using the Montgomery–Åsberg Depression Rating Scale (MADRS) [23] at baseline (*T*_0_) and at the end of hospitalization (*T*_1_). To describe the domains of depression, we also used Quilty’s factorial structure of MADRS [24], which comprises “*sadness*” (MADRS items 1 and 2), “*negative thoughts”* (Items 9 and 10), “*detachment*” (Items 6, 7, and 8), and the “*neurovegetative symptoms*” (Items 3, 4, and 5). At *T*_1_, a 50% reduction in the MADRS total score with respect to *T*_0_ [25] and a total score below 10 were defined as response and remission [26], respectively.

### Statistical analysis

Data management and descriptive analyses were conducted using the software Jamovi [27]. Network analysis was performed using R 4.3.1 [28]. We assessed the distribution using the Shapiro–Wilk test. Mann–Whitney, Wilcoxon rank-sum test, and *χ*^2^ test were used to test differences with a significant threshold of *p*-value = 0.05. We also stratified the sample based on the outcome at discharge: *very much improvement* (VMI; ≥80% decrease of MADRS total score at *T*_1_) and *low improvement* (LI; <80% decrease of MADRS total score at *T*_1_) [25] and compared MADRS item scores between samples.

### Network analysis

The network structure was estimated and visualized using “*qgraph*” [29], and its precision was evaluated using the “*bootnet*” [30]. Multiple network analyses of symptoms were calculated: first, an analysis of the overall sample at *T*_0_ and *T*_1_; second, symptom data at *T*_0_ were stratified for the clinical outcome at *T*_1_ (VMI vs. LI); third, separate analyses were performed for each diagnosis (BD and MDD). Each MADRS item was considered a node, and the edges between nodes represented their relationship, estimated using partial correlation. A covariance matrix was constructed on MADRS scores, and the network models were estimated using Spearman’s correlations. We chose the least absolute shrinkage and selection operator regularization technique [31, 32] as an estimator, and it was employed to retrieve sparse networks. We set the extended Bayesian Information Criterion hyperparameter, *γ*, according to the *netSimulator* results (0.5). This tool allows us to determine the *γ* value required to successfully examine a specific network structure in terms of sensitivity, specificity, correlation, strength, and closeness. The network structure was defined by the following network centrality measures [33]: strength, closeness, and betweenness of different nodes (or depressive symptoms) [30, 34]. Strength is the sum of edge weights directly connected to other nodes and represents the overall importance of a symptom in the network. Closeness is the inverse of the average shortest path length between a node and other nodes and measures how close the link is between the symptom and the others. Betweenness is the number of times that the node lies within the shortest path between two other nodes. We compared differences in global network structure and global strength (overall connectivity level by average strength of all edge weights) between time points or response groups using the “*Network Comparison Test*,” a two-tailed permutation test with 5,000 iterations [15, 35]. A *p*-value < 0.05 indicated a significant difference. We also evaluated the network accuracy and stability of the centrality indices (CS-coefficient) using “*bootnet.*” The accuracy of the edge weights was measured by the 95% confidence intervals obtained from 5,000 bootstrap samples drawn from the study population. The CS-coefficient quantifies the maximum proportion of cases that can be dropped at random to retain, with 95% certainty, a correlation of at least 0.7 with the centralities of the original network. The CS coefficient should be greater than and ideally above 0.50 [34]. Furthermore, the contribution of each edge within the symptom network associated with positive clinical outcomes to ECT was ranked using the relative risk (RR) to differentiate VMI relative to LI (see Supplementary Materials for details).

## Results

### Descriptive statistics of depressive symptoms and characteristics of participants

A total of 154 patients with DTD (121 MDD and 34 BD; 64.37% female) with a mean age of 55.3 years (SD = 12.8) were included. The average duration of the current hospitalization was 37.91 days (SD = 15.12) (see Table 1 for details of the sample characteristics).

**Table 1.** Sample characteristics: *Z* = Mann–Whitney *U*

Variables	Total	Very much improvement (*n* = 69)	Low improvement (*n* = 85)	*Z*/*χ*^2^	p
Age (y), mean (SD)	55.34 (12.75)	54.17 (12.96)	56.28 (12.57)	2688	0.375
Sex, *n* Females (%)	99 (64.3%)	38 (55.1%)	61 (71.8%)	4.62	0.032
Education, mean (SD), years	9.35 (4.18)	9.57 (4.09)	9.18 (4.27)	2711	0.404
Age of onset, mean (SD), years	34.33 (13.24)	34.22 (14.39)	34.42 (12.34)	2027	0.777
Diagnosis, *n* (MDD%)	121 (78.6%)	51 (73.9%)	70 (82.4%)	1.61	0.204
Recurrence, *n* (%)	130 (84.4%)	54 (78.3%)	76 (89.4%)	3.60	0.058
Psychotic symptoms, *n* (%)	99 (64.3%)	39 (56.5%)	60 (70.6%)	3.28	0.070
Comorbidities					
Psychiatric, *n* (%)	86 (55.8%)	33 (47.8%)	53 (62.4%)	3.26	0.071
Personality disorder, *n* (%)	47 (30.5%)	18 (26.1%)	29 (34.1%)	1.16	0.282
Anxiety disorders, *n* (%)	46 (29.9%)	18 (26.1%)	28 (32.9%)	0.854	0.355
Alcohol use disorder, *n* (%)	3 (1.9%)	2 (2.9%)	1 (1.2%)	0.591	0.442
Medical, *n* (%)	60 (39.0%)	24 (34.8%)	36 (42.4%)	0.918	0.338
Drug treatment					
APS (%)	59.1	53.6	63.5		
AD (%)	84.4	89.9	80.0		
MS (%)	31.2	29.0	32.9		
BDZ (%)	95.5	97.1	94.1		

The MADRS total score improved significantly at the endpoint (*T*_0_ = 33.03 [SD = 6.32]; *T*_1_ = 8.27 [SD = 5.98]; *p* < 0.05; Table 2]. Except for the MADRS total score at *T*_0_, normality was not met. A total of 145 (94.1%) participants achieved a response, and 98 (63.6%) participants remitted at *T*_1_.

**Table 2.** MADRS item scores at *T*_0_ and *T*_1_. Wilcoxon rank-sum test

MADRS item #	Symptom	*T*_0_ (Mean ± SD)	*T*_1_ (Mean ± SD)	p
Sadness		9.23 (1.77)	2.08 (2.08)	< .001
1	Apparent sadness	4.62 (1.01)	1.06 (1.04)	< .001
2	Reported sadness	4.62 (0.95)	1.03 (1.14)	< .001
Neurovegetative symptoms		8.36 (3.26)	1.76 (2.21)	< .001
3	Inner tension	4.25 (1.18)	1.04 (1.09)	< .001
4	Reduced sleep	2.42 (1.74)	0.53 (1.28)	< .001
5	Reduced appetite	1.73 (1.85)	0.19 (0.63)	< .001
Detachment		12.23 (2.84)	3.77 (2.60)	< .001
6	Concentration difficulties	3.84 (1.51)	1.27 (1.08)	< .001
7	Lassitude	4.30 (0.98)	1.40 (1.11)	< .001
8	Inability to feel	4.11 (1.10)	1.10 (1.03)	< .001
Negative thoughts		3.17 (1.82)	0.66 (0.90)	< .001
9	Pessimistic thoughts	2.77 (1.62)	0.58 (0.88)	< .001
10	Suicidal thoughts	0.40 (0.73)	0.07 (0.26)	< .001
Total score		33.03 (6.32)	8.27 (5.98)	< .001

Within the whole sample, 69 (44.8%) participants were classified as VMI (Table 3). At *T*_0_, VMIs had significantly greater severity in the d*etachment* domain (*p* = 0.021) and s*uicidal thoughts* (*p* = 0.010), and a trend for greater severity for *concentration difficulties* (*p* = 0.059) and i*nability to feel* (*p* = 0.088) and reduced severity for “i*nner tension*” (*p* = 0.025) and relative to LI.

**Table 3.** *T*_0_ MADRS items scores stratified by overall depression improvement, “very much improvement” (≥80%) versus those with “low improvement” (<80%)

MADRS item #	Symptom	Very much improvement (*n* = 69)	Low improvement (*n* = 85)
Sadness		9.45 (1.85)	9.06 (1.69)
1	Apparent sadness	4.71 (1.09)	4.54 (0.93)
2	Reported sadness	4.74 (0.97)	4.52 (0.93)
Neurovegetative symptoms		8.42 (3.42)	8.31 (3.15)
3	Inner tension*	4.09 (1.13)	4.39 (1.20)
4	Reduced sleep	2.52 (1.73)	2.34 (1.76)
5	Reduced appetite	1.91 (1.92)	1.58 (1.79)
Detachment*		12.81 (2.79)	11.75 (2.81)
6	Concentration difficulties§	4.16 (1.34)	3.59 (1.60)
7	Lassitude	4.41 (0.90)	4.21 (1.05)
8	Inability to feel	4.25 (1.18)	4.00 (1.03)
Negative thoughts		3.17 (1.96)	3.17 (1.70)
9	Pessimistic thoughts	2.61 (1.58)	2.89 (1.64)
10	Suicidal thoughts*	0.57 (0.85)	0.27 (0.59)
Total score		33.96 (6.54)	32.28 (6.06)

Statistic: *p < 0.05; §*p* = 0.059. Mann–Whitney *U* test.

### Network analyses

**Figure 1.**
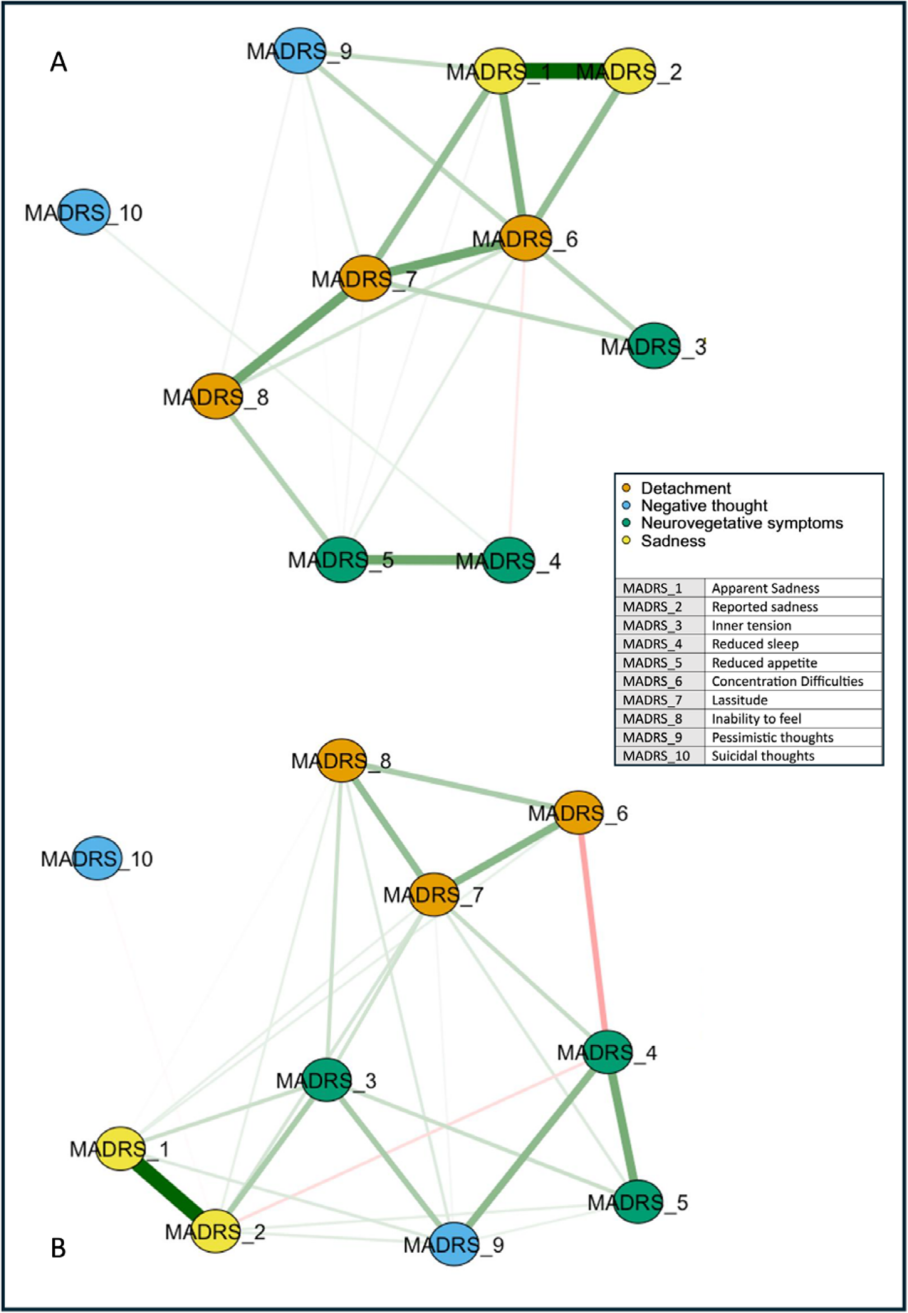
Networks of depressive symptoms in patients with difficult-to-treat depression before and after ECT. Network showing the associations among the MADRS item nodes (within circles) at *T*_0_ (A) and *T*_1_ (B). The color of the MADRS items represents Quilty’s factorial structure domains of the MADRS. Green lines (edges) indicate positive (partial) associations; red edges indicate negative associations. The thickness of the lines indicates the strength of the associations. Quilty’s domains and MADRS items are indicated in the inset.

**Figure 2.**
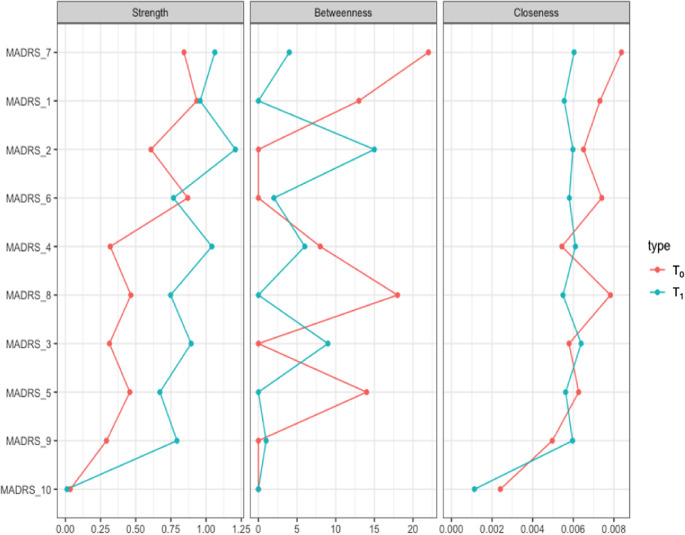
Centrality measures of the study variables at *T*_0_ (in red) and *T*_1_ (in blue) in the whole sample. On the *x*-axis: the value of the centrality measures (strength, betweenness, closeness). On the *y*-axis: MADRS items (nodes).

We represented the networks of the overall sample at *T*_0_ and *T*_1_ (Figure 1). Network global strength increased significantly at *T*_1_ relative to *T*_0_ (*p* = 0.03). Moreover, the edge strength of the positive relationships *reduced sleep*–*pessimistic thoughts*, *inner tension*–*reduced appetite*, and *reduced sleep*–*lassitude*, and the negative relationship *reduced sleep*–*concentration difficulties* increased at *T*_1_ relative to *T*_0_ (Figure 2). Betweenness was increased for *reported sadness* (*p* = 0.05) and reduced for *inability to feel* at *T*_1_ relative to *T*_0_ (*p* = 0.05).

The diagnosis subgroup analyses identified topological patterns similar to the overall sample. In particular, the overall symptom network structure, but not the strength of the global network, was significantly affected in BD (*p* = 0.004) and marginally in MDD (*p* = 0.072) by ECT.

**Figure 3.**
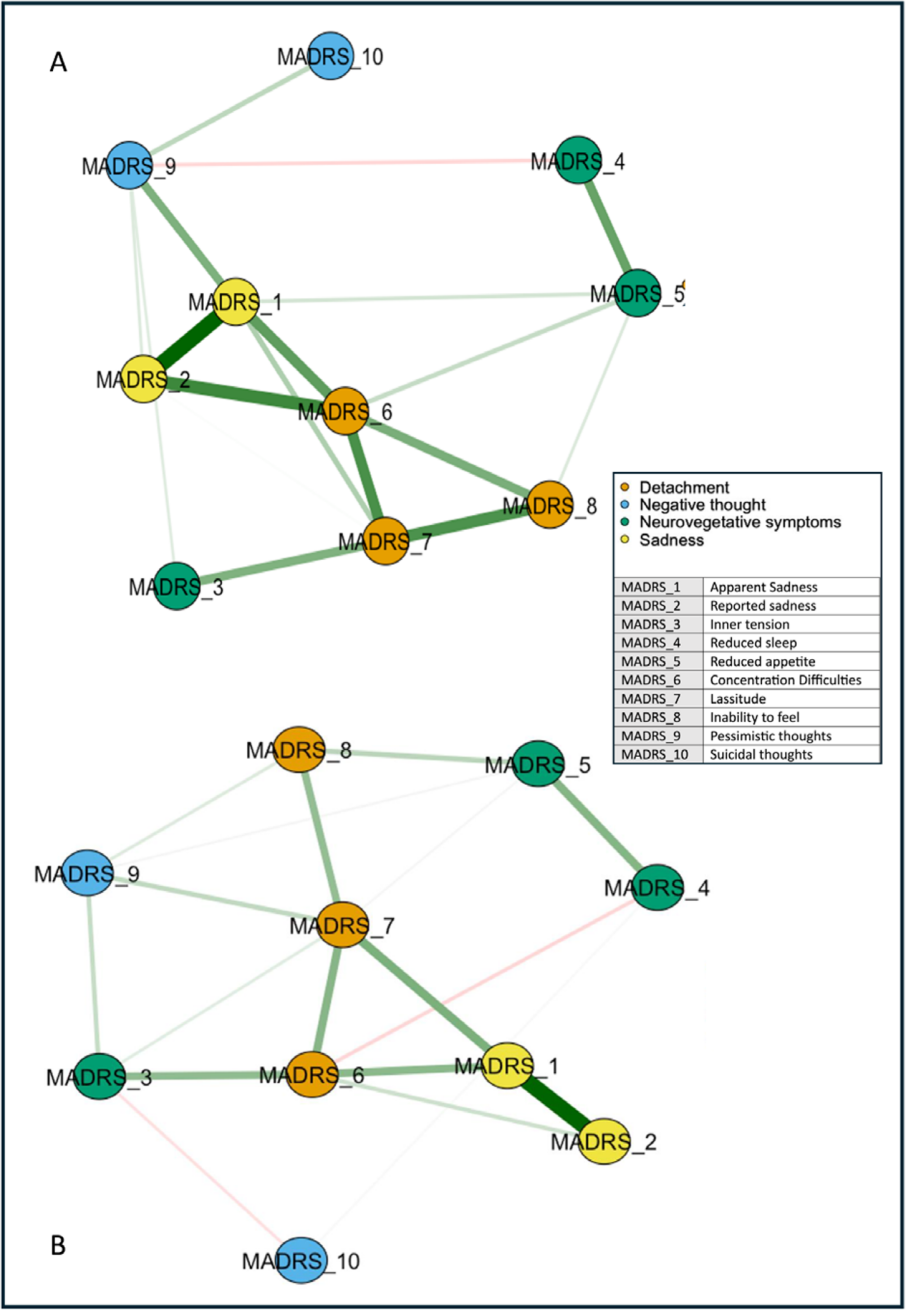
Networks of depressive symptoms in patients with difficult-to-treat depression at *T*_0_ stratified by outcome. LI, low improvement; VMI, very much improvement. Network showing the associations among the MADRS items nodes (within circles) in patients with VMI (A) and LI (B). The color of the MADRS items represents Quilty’s factorial structure domains of the MADRS. Green lines (edges) indicate positive (partial) associations; red edges indicate negative associations. The thickness of the lines indicates the strength of the associations. Quilty’s domains and MADRS items are indicated in the inset.

**Figure 4.**
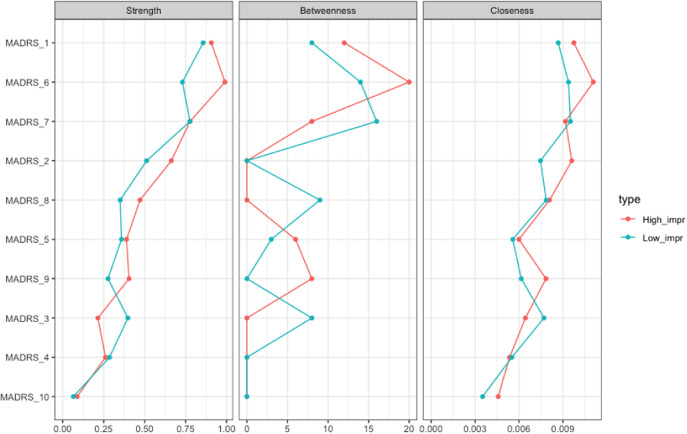
Centrality measures of the study variables stratified by outcome, “very much improvement” (in red) versus “low improvement” (in blue) at *T*_0_. On the *x*-axis: the value of the centrality measures (strength, betweenness, and closeness). On the *y*-axis: MADRS items (nodes).

The network analysis stratified for the response showed no difference in global networks of VMI and LI at *T*_0_ (*p* = 0.809; Figure 3). Edgewise analyses showed a greater negative *reduced sleep*–*pessimistic thoughts* relationship (*p* = 0.047) and a trend of significance for the positive *pessimistic thoughts*–*suicidal thoughts* relationship (*p* = 0.06) in VMI compared to LI. VMIs had the greatest centrality (strength) for *concentration difficulties*, closeness for *apparent sadness*, and *concentration difficulties*, and betweenness for *apparent sadness*, *concentration difficulties*, *lassitude*, and *pessimistic thoughts*, respectively (Figure 4). *Apparent sadness* had the greatest centrality in LIs. Centrality measures did not differ between groups.

### RR for outcome prediction (VMI vs. LI)

The edge with the highest RR for VMI was *inner tension*–*lassitude* (logRR = 2.098) and *reduced sleep*–*pessimistic thoughts* (logRR = 1.723). The edge *reported sadness*–*suicidal thoughts* showed the lowest RR (logRR = 0.064). Detailed results are reported in Supplementary Table S.1.

## Discussion

In this study, we found that the network of depressive symptoms was modulated by ECT, with increased strength of the global network and the edges between somatic (appetite and sleep) and cognitive-emotional symptoms (tension, lassitude, and pessimistic thoughts). In patients with VMI, *pessimistic thoughts* had a greater negative association with *reduced sleep* and *suicidal thoughts* relative to LI, with the greatest strength for *concentration difficulties* and *apparent sadness* for VMI and the greatest centrality for *apparent sadness* in LIs. Within the symptom network associated with the outcome, the strength of the edges *inner tension-lassitude* and *reduced sleep*-*pessimistic thoughts* showed the highest contribution.

The increase in global strength following ECT is consistent with previous studies showing a similar effect for antidepressant drugs [36, 37], including paroxetine [38] and other SSRIs [36, 37] in MDD. According to the network theory [9], when the relationship between symptoms becomes weaker, the likelihood of one symptom eliciting another decreases. Thus, network connectivity is expected to decrease as symptoms ameliorate. This property has been proven only in cross-sectional between-subject investigations [39]. Our study confirmed this result, extending to the longitudinal changes in the relationship between symptoms. The increase in global strength suggests that depressive symptoms became more strongly interconnected after treatment, so that changes in one symptom were more likely to influence others. This could suggest greater coherence or unity among symptoms after ECT, potentially reflecting changes in the way the brain processes or experiences depressive symptoms as a whole [40].

ECT appeared to modify the interaction between neurovegetative symptoms. The literature shows that this cluster improves rapidly during ECT sessions [41, 42]. Notably, the improvement of symptoms in the neurovegetative domain (appetite symptoms, psychomotor symptoms, and hypersomnia) and suicidal ideation was shown to precede the improvement of mood symptoms after ECT [43]. Our findings expand on this result, showing that with ECT treatment, the network of symptoms undergoes a reconfiguration mainly in the neurovegetative domain (reduced sleep and reduced appetite). We also found low centrality measures in this domain [17], indicating a minor importance in the network of symptoms due to a reduced relationship with other nodes. We can hypothesize a later appearance with respect to the symptoms with measures of greater centrality (sad mood or fatigue). Symptoms with low centrality may either emerge later in the course of the depressive episode, as they are less influenced by other nodes, or remit earlier, given their limited integration within the symptom structure. These hypotheses are consistent with network-based models of depression, which conceptualize highly central symptoms as potential drivers of symptom maintenance and spread [17]. Consistently, previous studies on the effects of ECT on somatic symptoms have shown that sad mood could be the last symptom to show improvement as a result of the amelioration of neurovegetative symptoms [42, 44, 45]. Based on this finding, the magnitude of the relationship between *inner tension* and *lassitude* was associated with a greater likelihood of response to ECT. In addition, the relationship between *pessimistic thoughts* and *reduced sleep* and between *apparent sadness* and *pessimistic thoughts* predicted a better response to ECT, thus suggesting that tighter connectivity in the core affective-cognitive domain could be linked with the effectiveness of this treatment. Overall, these findings could support a better response to ECT in melancholic depression [46]. In contrast, the dyad between *reported sadness* and *suicidal thoughts* showed the lowest contribution within the symptom network, indicating a reduced importance of this connection to better outcomes. This relative disconnection could suggest a more differentiated and modular symptom structure, potentially less malleable to the effects of neuromodulation. This interpretation aligns with previous research suggesting that suicidality can follow an autonomous trajectory [43], and that its reduction often precedes mood improvement during ECT [42, 44, 45]. The finding that suicidal thoughts are less centrally anchored to depressive symptoms in patients with better outcomes may contribute to explaining the early and more substantial reduction in suicidality observed in responders BD [47–52] and could be explored as a network-level marker of treatment nonresponse.

ECT also increased betweenness for *reported sadness* but reduced it for the *inability to feel.* This finding aligns with the evidence for earlier detection of patients’ clinical improvement by clinicians and eventually by patients themselves [53]. However, betweenness is considered unstable in cross-sectional and temporal networks, which warrants further confirmation [34].

In patients with better outcomes, *pessimistic thoughts* had a more negative association with *reduced sleep* and a more positive association with *suicidal thoughts* at baseline. This is further supported by the higher likelihood of response associated with the strength of the relationship between *sleep* and *pessimistic thoughts*, suggesting a strong interrelationship that may serve as a prognostic factor.

Our study underscores the clinical importance of the *negative thoughts* cluster. In particular, suicidality was a strong symptom within the network, with lower measures of centrality. This is in line with the literature on MDD [43] showing that suicidal ideation amelioration after ECT anticipated improvements in mood symptoms and had an independent trajectory. Furthermore, other studies found that suicidality is more likely to influence other symptoms [54–56]. The severity of insomnia is a well-known predictor of suicidal ideation during depression [57], and negative thoughts can contribute to the development and maintenance of sleep disturbances [58]. Notably, ECT treatment has been shown to be effective in reducing suicidality, including suicidal ideation and suicide attempts in MDD and BD [47–52]. The strong connection between *reduced sleep* and *pessimistic thoughts* can reflect a bidirectional link, in which insomnia and negative ruminations can enhance each other. The prominence of this connection in patients with better outcomes suggests it may function as a “bridge” between the somatic and cognitive domains, enhancing ECT effectiveness. Thus, the relationship between reduced sleep and pessimistic thoughts may serve as a marker of the ECT-sensitive network topology.

Previous research [12, 35] has shown that treatment outcomes may be affected by central symptoms. In DTD, with better outcomes after ECT, concentration difficulties and apparent sadness were the central symptoms compared to patients with worse outcomes, in whom lassitude and apparent sadness were central in the network. The centrality of the apparent sadness node at baseline is consistent with other studies, in which the mood cluster showed greater improvement compared to other symptom clusters (including sleep and anxiety) during ECT [8, 59].

Previous studies have highlighted the importance of mood symptoms in the description of depression [17]. The presence of central symptoms can be related to the maintenance of the symptomatology itself [11]. Central symptoms, including fatigue and depressed mood, are considered principal symptoms in the DSM [19] and could contribute to the persistence of depressive conditions [16, 17]. Particularly, depressed mood was identified as a driving symptom that may activate other symptoms [18]. Therefore, the presence of apparent sadness at baseline as a central symptom in the networks in patients, regardless of their outcome to ECT, is consistent with the observation that no predictive network structure for the best response could be identified within our sample. Conversely, a recent study identified suicidal ideation, psychomotor retardation, and hypochondriasis as predictors of remission in severe depression using network analysis [60]. Dissimilarities in the patients (DTD vs. MDD), outcome (remission vs. response levels), and assessment tools (MADRS vs. Hamilton Rating Scale for Depression) limited the comparability of their results with ours due to potential differences in terms of neurobiology [61], outcome, and symptom structure [62].

Lassitude and fatigue are described as highly central in many studies [63, 64] and are also hypothesized as potential nodes for the onset of depression [65]. Malgaroli et al. [17] performed a systematic review of the literature on the networks of symptoms in MDD. They highlighted that in 23 studies, fatigue (i.e., lassitude) often had a higher strength centrality compared to cognitive symptoms that ranked in the middle. Sleep disturbances and suicidal ideation were found to have low centrality measures. These findings were also confirmed by our study. Furthermore, a recent study that investigated the effects of Stanford Neuromodulation Therapy in treatment-resistant depression found that lassitude could predict the outcome. More specifically, the authors hypothesized that a reduced lassitude could ameliorate depressive symptoms because patients can start reengaging in their daily routine, similar to behavioral activation strategies [66]. Notably, the depressive symptoms modulated by ECT, including sleep problems, fatigue, and cognition, closely overlap with those that have the greatest impact on quality of life and are considered key targets for the treatment of DTD [20].

We must acknowledge some limitations. First, it is unclear whether network modifications are due to the improvement of symptoms or the application of ECT itself. Second, ECT administration varied across patients with differences in terms of electrode placement and the number of sessions. Unfortunately, we did not have sufficient power to perform a stratification analysis for these parameters. Fourth, patients had psychiatric and physical comorbidities, which could influence the network structure and the response to ECT. In addition, we acknowledge that the generalizability of our findings may be limited by the specific features of our DTD sample, including older age, longer duration of the disease, greater recurrence, medical and psychiatric comorbidity, longer duration, and more complex treatments [67–69]. However, these characteristics are more reflective of the patients who undergo ECT treatment. Lastly, given the retrospective design of our study, potential biases, such as selection or information bias, cannot be entirely ruled out [70]. Nonetheless, to mitigate their effects, we used a thorough and accurate collection of the data, keeping in account potential nuisance variables, and used standardized and objective rating scales. Future prospective studies with larger, more diversified samples and standardized treatment protocols are needed to confirm and expand on these findings. In particular, longitudinal studies should investigate whether network changes persist beyond the immediate posttreatment phase. Experimental designs with controlled comparisons between different treatment modalities, such as pharmacotherapy, psychotherapy, or other neuromodulation techniques, could help clarify the causal relationships between symptom connectivity changes and therapeutic response. In addition, further exploration of the predictive value of specific symptom networks could enhance personalized treatment strategies for individuals with DTD.

In conclusion, ECT can modulate not only the severity of symptoms but also their relationship, and this may contribute to the clinical response in DTD. Future studies addressing the specificity of these results are warranted to help understand the association of these changes with the brain mechanisms hypothesized for ECT.

## Supporting information

10.1192/j.eurpsy.2025.10052.sm001 Lussignoli et al. supplementary material Lussignoli et al. supplementary material

## Data Availability

The data that support the findings of this study are available upon reasonable request from the corresponding author.

## Supplementary material

The supplementary material for this article can be found at http://doi.org/10.1192/j.eurpsy.2025.10052.
