# Electroconvulsive therapy modulates the interplay between depressive symptoms in difficult-to-treat depression: A longitudinal network analysis – CORRIGENDUM

**DOI:** 10.1192/j.eurpsy.2025.10080

**Published:** 2025-09-23

**Authors:** Marialaura Lussignoli, Marco Bortolomasi, Giulia Perusi, Giorgio Pigato, Alessandra Minelli, Fabio Sambataro

**Keywords:** Bipolar disorders, Difficult-to-treat depression, Electroconvulsive therapy, Major Depressive Disorder, network analysis, corrigendum

This article was originally published with an error in the funding statement. It initially read as follows:

The assistant research position of Giorgio Pigato is funded by ERA-PerMed PROMPT project [IT-MoH ERP-2020-23671059].

In fact, it was the assistant research position of author Giulia Perusi which was funded by ERA-PerMed PROMPT project [IT-MoH ERP-2020-23671059].

This has now been amended in the article and this corrigendum published.
