# Supplementary material for: Electroconvulsive therapy modulates the interplay between depressive symptoms in difficult-to-treat depression: A longitudinal network analysis
Source: Eur Psychiatry. 2025 Jun 16;68(1):e84. doi: 10.1192/j.eurpsy.2025.10052 (PMC12260723; doi:10.1192/j.eurpsy.2025.10052)

Supplementary materials

**Electroconvulsive Therapy modulates the interplay between depressive symptoms in Difficult to Treat Depression: a longitudinal network analysis**

M. Lussignoli ^1,2^, M. Bortolomasi ^3^, G. Perusi ^4^, G. Pigato ^5^, A. Minelli ^4,6^, F. Sambataro ^1,2^

^1^ Department of Neuroscience (DNS), University of Padova, Padua, Italy

^2^ Padova Neuroscience Center, University of Padova, Padua, Italy

^3^ Psychiatric Hospital “Villa Santa Chiara”, Verona, Italy

^4^ Department of Molecular and Translational Medicine, University of Brescia, Brescia, Italy

^5^ Padua University Hospital, Padua, Padua, Italy

**^6^ Genetics Unit, IRCCS Istituto Centro San Giovanni di Dio Fatebenefratelli, Brescia, Italy**

[Supplementary Figure 1. Relative Risks (RR) of Symptom Connections (Baseline) 3](#_Toc196817594)

[Supplementary Table 1. Ranking of Connections by Relative Risk (RR) 4](#_Toc196817595)

*Ranking of edges Relative Risk to quantify the outcome prediction (VMI vs LI):*

**Supplementary Methods**

We quantitatively ranked the weight of the edges using Relative Risk (RR) of positive outcome, comparing VMI and LI. This analysis allowed us to estimate the likelihood of specific symptom connections emerging in one group compared to the other (*VMI* vs. *LI*), as well as to inform predictions about clinical outcomes and response. To investigate these relative risks more thoroughly, symptom networks were estimated at baseline for both groups. We employed the partial correlation (pcor) estimator, as it permits the construction of denser networks without the use of regularization, thereby facilitating a more detailed and nuanced analysis of the associations between individual symptom pairs.)

**Supplementary Results**

We examined the Relative Risk (RR) to assess the weight of specific edges in the symptom network when comparing VMI and LI following ECT. This metric reflects how much more likely a given connection between two symptoms will occur in one group relative to the other. Full results are reported in *Supplementary Table 1.* Edges with RR greater than 1 indicate stronger associations among symptoms in the VMI group, potentially representing markers of a favorable treatment response. Conversely, RR below 1 highlights the edges that are more prominent in the LI group, suggesting symptom interactions that may underlie treatment resistance.

The edge with the highest RR was observed between *inner tension* and *lassitude* (log RR = 2.098), indicating a notably stronger connection in high responders. Similarly, the link between *reduced sleep* and *pessimistic thoughts* was more pronounced in VMI (log RR 1.723), further supporting its relevance as a potential predictor of ECT response. On the other end, edges with lower RR values, detailed in Supplementary Table 1, characterize symptom associations more common among low responders.

# **Supplementary Figure 1.** Relative Risks (RR) of Symptom Connections (Baseline)


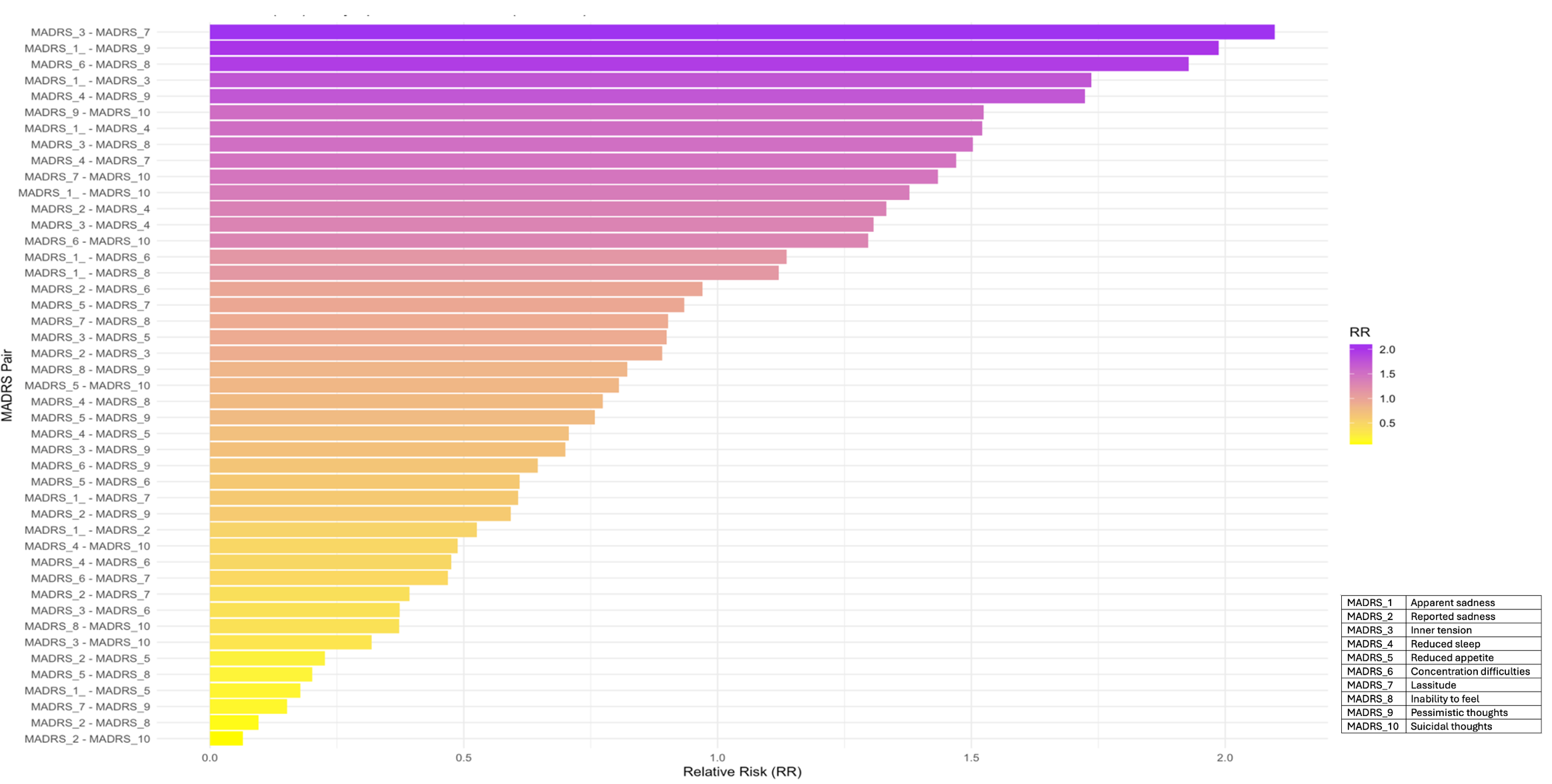


# **Supplementary Table 1.** Ranking of Connections by Relative Risk (RR)


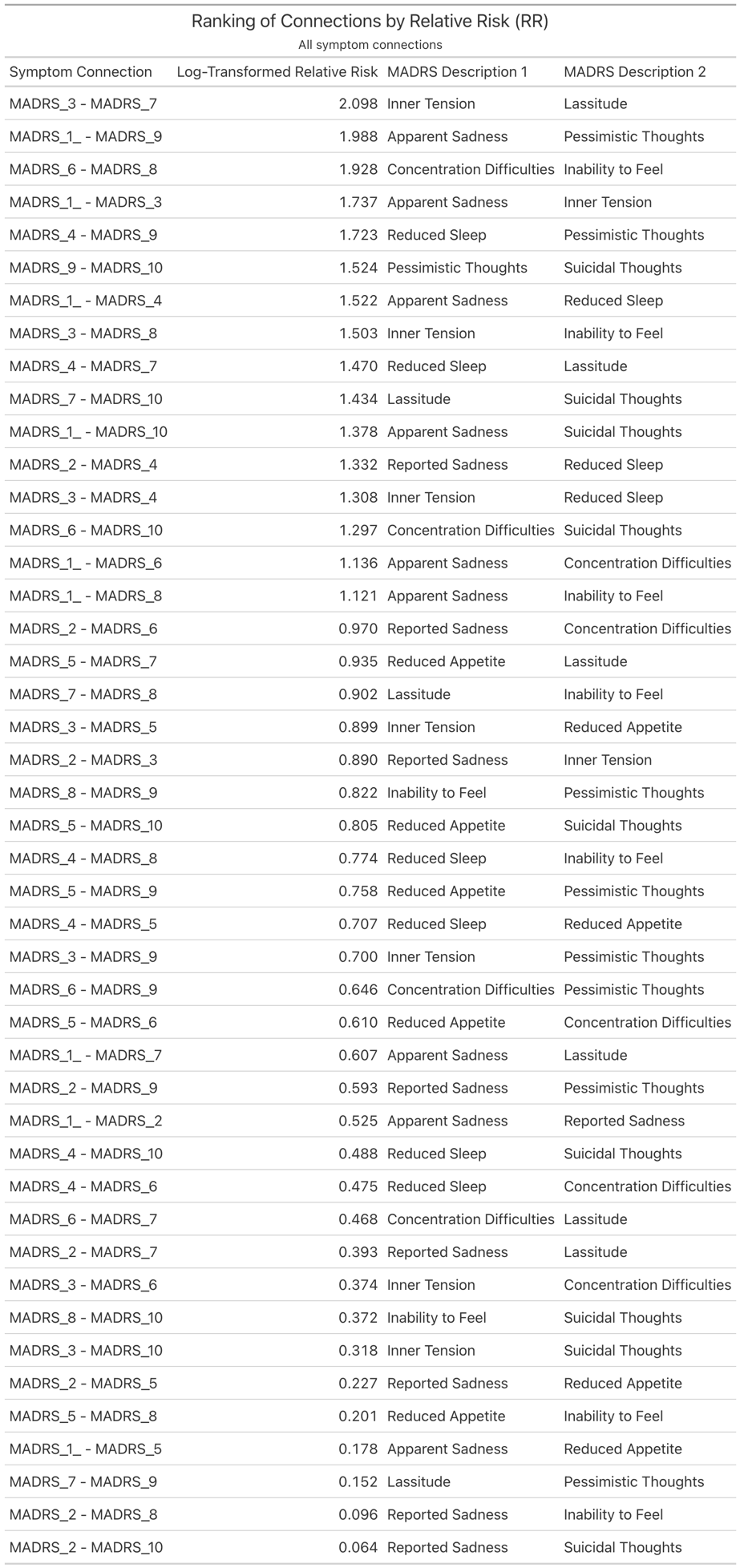

Supplement: Lussignoli et al. supplementary material [file S0924933825100527sup001.docx]
